# Genetic assessment and candidate genes identification for breed-specific characteristics of Qingyuan partridge chicken based on runs of homozygosity

**DOI:** 10.1186/s12864-024-10492-y

**Published:** 2024-06-10

**Authors:** Xing Zhang, Liu Yang, Zhuojun Xie, Jiankang Gan, Piao Zhu, Jiani Song, Huimin Kang, Zhengfen Zhang, Lingbin Liu, Hai Xiang, Hua Li

**Affiliations:** 1 Guangdong Provincial Key Laboratory of Animal Molecular Design and Precise Breeding, School of Life Science and Engineering, Foshan University, Foshan, 528225 China; 2 Key Laboratory of National Forestry and Grassland Administration on Conservation Biology of Rare Animals in the Giant, China Conservation and Research Centre for the Giant Panda, Panda National Park, Chengdu, 611830 China; 3 Guangdong Tinoo’s Foods Group Co., Ltd, Qingyuan, 511827 China; 4 College of Animal Science and Technology, Southwest University, Chongqing, 400715 China

**Keywords:** Chicken, Inbreeding coefficient, Runs of homozygosity, Candidate genes

## Abstract

**Background:**

Several core breeding and supporting lines of the Qingyuan partridge chicken, a representative local chicken breed in China, have been developed over 20 years. Consequently, its economic traits related to growth and reproduction have been significantly improved by breeding selection and commercial utilization, but some characteristic traits, such as partridge feathers, high meat quality and sufficient flavor, have always been retained. However, effective methods for genetic assessment and functional gene exploration of similar trait groups are lacking. The presence of identical haplotype fragments transmitted from parent to offspring results in runs of homozygosity (ROH), which offer an efficient solution. In this study, genomes of 134 Qingyuan partridge chickens representing two breeding populations and one preserved population were re-sequenced to evaluate the genetic diversity and explore functional genes by analyzing the diversity, distribution, and frequency of ROH.

**Results:**

The results showed a low level of genomic linkage and degree of inbreeding within both the bred and preserved populations, suggesting abundant genetic diversity and an adequate genetic potential of the Qingyuan partridge chicken. Throughout the long-term selection process, 21 genes, including *GLI3*, *ANO5*, *BLVRA*, *EFNB2*, *SLC5A12*, and *SVIP*, associated with breed-specific characteristics were accumulated within three ROH islands, whereas another 21 genes associated with growth traits including *IRX1*, *IRX2*, *EGFR*, *TPK1*, *NOVA1*, *BDNF* and so on were accumulated within five ROH islands.

**Conclusions:**

These findings provide new insights into the genetic assessment and identification of genes with breed-specific and selective characteristics, offering a solid genetic basis for breeding and protection of Qingyuan partridge chickens.

**Supplementary Information:**

The online version contains supplementary material available at 10.1186/s12864-024-10492-y.

## Background

Chickens are by far the most abundant domesticated animals and the preferred source of animal protein [1]. Since being domesticated for thousands of years [2–7], more than 107 local chicken breeds have been developed in China through long-term natural and artificial selection [8, 9], resulting in the development of individual characteristics associated with each breed. The selection sweep usually leaves a footprint on the genome, resulting in the emergence of long haplotypes, high-frequency derived alleles and highly differentiated alleles in the genome [10]. Functional genes encoding the characteristic traits of each breed can be identified by determining the traces left by selection at the genome-wide level. Furthermore, various methods have been used to identify the characteristic traits of different animal breeds, including fixation index (*F*_ST_), nucleotide diversity, cross-population extended haplotype homozygosity (XPEHH), and cross-population composite likelihood ratio (XPCLR) [11], through which the characteristic traits of geese [12], cattle [13], ducks [14] and other agricultural animals [15, 16] have been identified. However, most studies have focused on identifying phenotypic differences between populations, whereby detecting selection signals in breed showing similar phenotypic characteristics has become a great challenge.

Inbreeding is a necessary process in animal breeding. The increase of inbreeding coefficient and reduction in inbreeding-resultant genetic variability have impacted the animal breeding negatively [17]. Although pedigree information is commonly used to evaluate inbreeding [18], it has proven ineffective, as pedigree information-based inbreeding are usually lower than true inbreeding [19–21], whereas the ROH-based F estimate (*F*_*ROH*_) is considered one of the closest approximations to the true inbreeding coefficient [22, 23]. ROH refers to the continuous homozygosity genotype region in the genome [24] and is applied to the study of population structure and the historical analysis of cattle [25], chickens [26, 27], sheep [28] and other animals [29]. Most ROHs are formed by inbreeding [20]. Furthermore, the number, length, frequency and distribution of ROH provide abundant genetic information [30].

Under the influence of long-term artificial and natural selection, the formation and distribution of ROH fragments in the animal genome are affected by many factors, such as the mating system, direction of selection, population size and history of population formation [31, 32]. The long length of ROH have a common ancestor from the most recent generation, and the genetic recombination of multiple generations of random mating continuously splits the pieces into fragments of shorter length [33]. Further, ROH are not evenly distributed throughout the genome with high-frequency regions known as ROH islands, which were more likely to form in selected regions with higher homozygosity and lower genetic diversity [34, 35]. Therefore, ROH are an effective means for identifying characteristic traits and selected genes, especially in animal breeds that require the preservation of breed-specific characteristics.

As one of China’s most famous high-quality native chicken breeds, the Qingyuan partridge chicken possesses excellent genetic traits and has been selected as ‘Top ten outstanding livestock and poultry germplasm resources of China’. After more than 15 years of breeding for preservation and utilization, the researchers performed a systematic study of Qingyuan partridge chicken from various perspectives. Several key candidate genes and molecular markers for production traits of Qingyuan partridge chicken have been identified [36–41], which provide a basis for the systematic evaluation, breeding and utilization of Qingyuan partridge chicken’s variety resources as well as the extensive market application and promotion.

During the breeding process, the growth traits of the Qingyuan partridge chicken were selected while retaining their characteristic traits. This model presents an excellent opportunity to investigate the role of ROH in identifying trait and functional gene mining in agricultural animals using various generation populations. In this study, 134 Qingyuan partridge chickens from three different populations, representing a preserved population (Q8) and two populations of different breeding generations (K6 and K14), were genomic sequenced. By analyzing the diversity, distribution, and frequency of ROH, the genomic assessment on Qingyuan partridge chicken were performed and functional genes related to breed-specific and selected characteristics were determined. The study not only contribute to the evaluation of the inbreeding coefficient and breeding preservation status of Qingyuan partridge chickens, but also provide new insights for performing genetic assessment and identifying the genes of breed-specific or selected characteristics.

## Result

### Comparison on body weight and body size between the preserved and breeding populations

The 120-day-old body weight and body size records of the K14 and Q8 populations were adopted from the National Breeding and Conservation Farm for Qingyuan partridge chickens (Table 1). The statistical analyses showed that the mean body weights and shank circumference of the K14 population at 120 days of age were significantly greater than those of the Q8 population (*P* < 0.01). The body weights of K14 roosters and hens were 19.3% and 13.7% heavier than those of Q8 roosters and hens, respectively. However, the difference in shank length of both K14 roosters and hens was not significant (*P* > 0.05) compared to Q8 population. Meanwhile, the coefficients of variation (CV) for all body weight and body size indices of roosters and hens in K14 population were lower than those of the Q8 population.

**Table 1:** Comparison on body weight and body size between K14 and Q8 at Day 120

Gender	population	number	body weight (g)	CV	shank length (cm)	CV	shank circumference (cm)	CV
♂	K14	298	1897.6 ± 141.6^B^	7.46%	7.19 ± 0.26	3.62%	4.29 ± 0.18^B^	3.59%
	Q8	94	1590.0 ± 140.1^A^	8.81%	7.23 ± 0.34	4.71%	3.86 ± 0.16^A^	4.02%
♀	K14	1127	1328.4 ± 112.2^B^	8.54%	5.93 ± 0.27	4.48%	3.52 ± 0.12^B^	3.53%
	Q8	100	1168.6 ± 111.4^A^	9.53%	5.88 ± 0.28	4.85%	3.26 ± 0.14^A^	4.20%

CV, coefficients of variation. Different capital letters indicate highly significant differences (*P* < 0.01), and no letter indicates non-significant differences (*P* > 0.05)

### Sequencing and data quality

We obtained 105.9-364.9 M raw resequencing reads for each Qingyuan partridge chicken, with an average sequencing depth between 22.78× and 25.29× across populations, providing sufficient data for subsequent analysis. After mapping quality control, 11,862,445 high quality SNPs from 129 individuals were included in the phylogenetic and population analyses (Table 2).

**Table 2:** Quality of sequencing data and SNP statistics

population	*N*		raw reads	clean reads	alignment rate(%)	Average depth (×)	No. of SNPs
	♂	♀					
K6	12	19	192,264,388	190,770,809	99.55	22.84	10,027,296
K14	30	29	214,150,468	212,278,955	99.65	25.29	9,637,775
Q8	29	15	211,835,902	210,127,358	99.48	22.78	9,478,611

### Population structure of the three populations

**Fig. 1:**
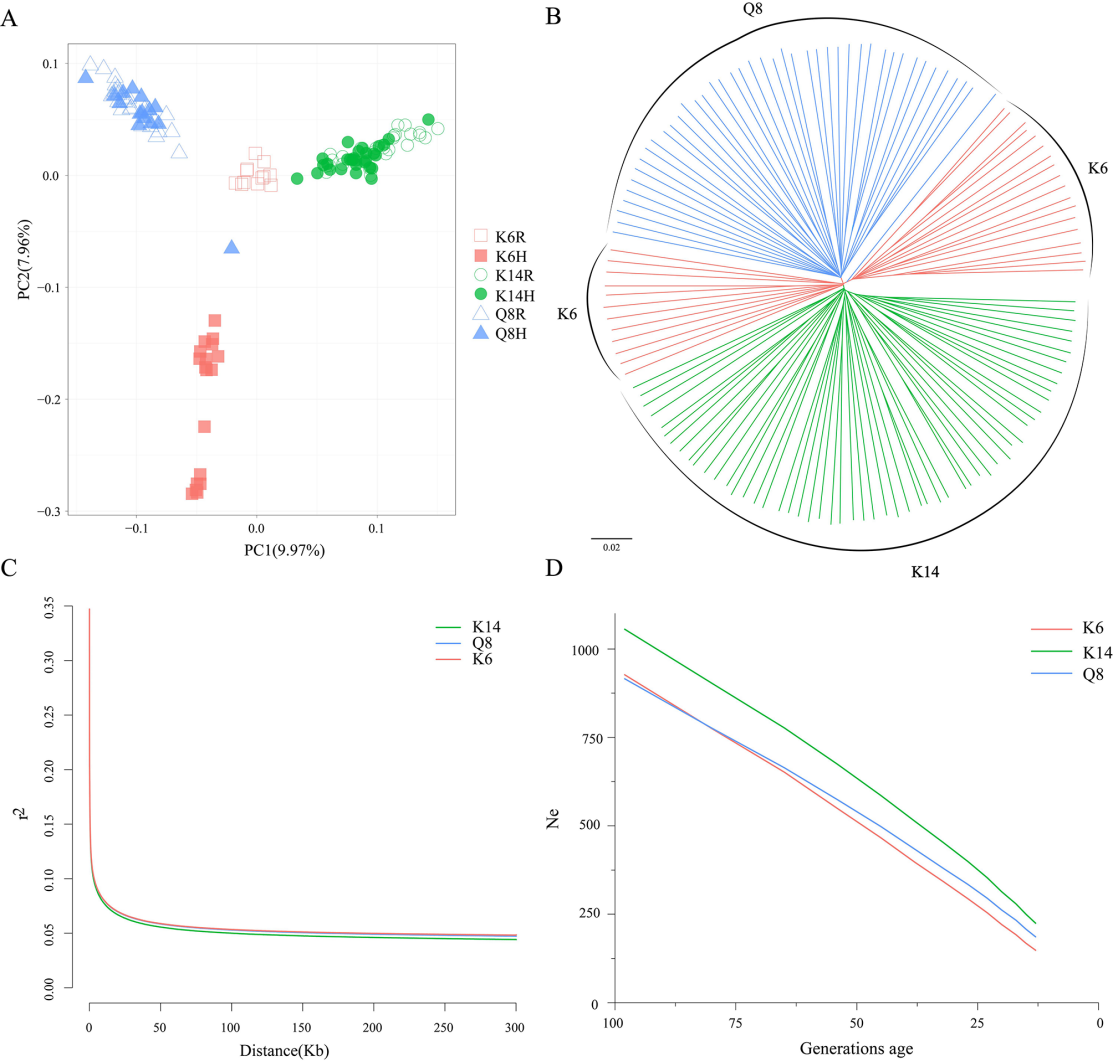
Genetic relationships, LD decay and historical generations effective population size of three populations of Qingyuan partridge chickens. (**A**) Principal component analysis of the 3 populations. Hollow and solid represent the rooster (R) and hen chickens (H) in the populations, respectively. (**B**) Neighbor-joining phylogenetic tree of 129 individual Qingyuan partridge chickens. (**C**) Linkage disequilibrium decay of three Qingyuan partridge chicken populations. (**D**) Historical generations effective population size of three Qingyuan partridge chicken populations

To infer the population structure of Qingyuan partridge chickens, principal component analysis and phylogenetic analysis were carried out using whole genome variation. Principal component analysis (PCA) showed that the three populations were clearly differentiated, with a high degree of aggregation of individuals within the same group (Fig. 1A). Interestingly, the males and females of the early selection population K6 were divided into two regions, which corresponded to the division of the K6 group into two major branches on the NJ tree (Fig. 1B), suggesting that the germplasm consistency of the early selection group K6 was insufficient, but gradually became consistent as the selection progressed (K14).

### Genetic diversity of the three populations

The early breeding population K6 had more abundant SNPs than the preserved population Q8 and the recent breeding population K14. However, the expected heterozygosity (He) and observed heterozygosity (Ho) were similar among the three populations. Moreover, the low inbreeding coefficients (F) for all three populations (about 0.01) also suggested high genetic diversity in both the preserved and breeding populations of Qingyuan partridge chickens (Table 3). When the physical distance reached 50 kb, the average value of the LD coefficient of the three populations was less than 0.1, and the LD coefficients decreased to about 0.05 when the physical distance was 100 kb. Overall, all three populations showed similarly weak genomic linkage disequilibrium (Fig. 1C). Furthermore, trends in effective population size based on linkage disequilibrium showed that the historical effective population size of the K14, K6, and Q8 populations gradually decreased from the 100th generation (Fig. 1D).

**Table 3:** Genetic diversity of three Qingyuan partridge chicken populations

Populations	SNPs	He	Ho	*F*
K6	10,027,296	0.2788	0.2820	0.012 ± 0.020
K14	9,637,775	0.2715	0.2771	0.009 ± 0.016
Q8	9,478,611	0.2734	0.2774	0.013 ± 0.018

*F*, inbreeding coefficient; *He*, expected heterozygosity; *Ho*, observed heterozygosity. Different lowercase letters indicate significant differences (*P* < 0.05) and no letter indicates non-significant differences (*P* > 0.05)

### ROH detection in the breeding and preserved populations

**Fig. 2:**
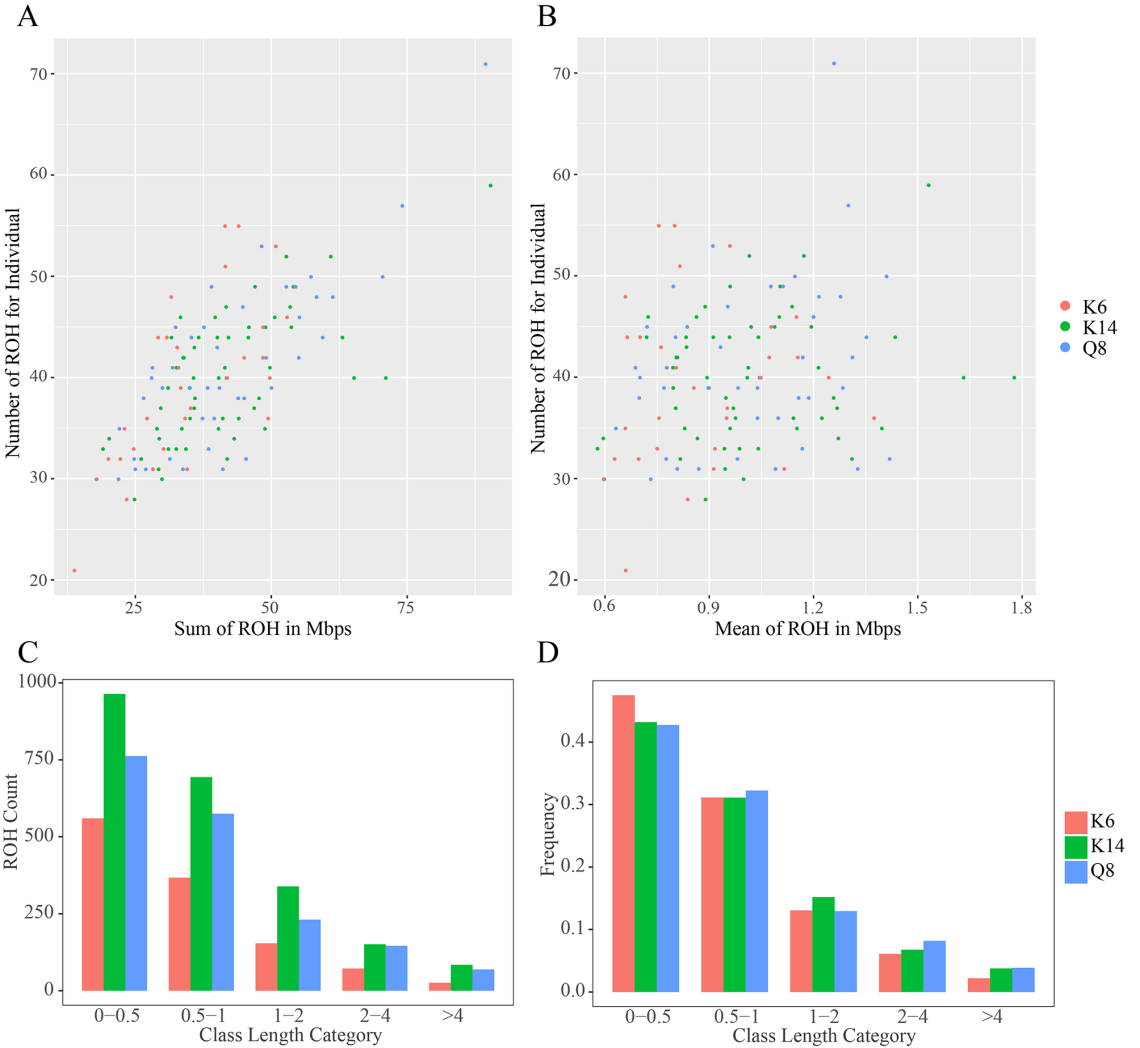
The statistical information of ROH. (**A**) and (**B**) Distribution of the total and average lengths of different individual ROHs and the total number of ROHs. (**C**) and (**D**) Number and percentage of ROH in different length categories

After removing redundant linkage sites by linkage disequilibrium pruning, 1,167,649 SNPs were obtained from the three populations and were submitted to ROH analysis. As a result, 5,194 ROH in total were detected among the three populations, 1,179 ROHs of which were detected in K6, 2,232 in K14, and 1,783 in Q8. Most individuals exhibited a range of 30–55 ROH fragments, while the total length of ROH in individuals from the three groups predominantly fell within the range of 20–60 Mb (Fig. 2A). Additionally, the average length of ROH in individuals varied from 0.6 to 1.5 M (Fig. 2B). Notably, there was an absence of identifiable subgroup clustering among individuals from the three groups based on the overall and mean lengths of ROH. However, it is worth noting that K6 exhibited smaller average and total ROH lengths than the Q8 and K14 populations. In each of the three populations, 44.0% of the ROHs had lengths ranging from 0 to 0.5 Mb. Additionally, the proportions of ROH with lengths of 0.5-1 Mb, 1–2 Mb, 2–4 Mb, and greater than 4 Mb were 31.5%, 13.9%, 7.1%, and 3.5%, respectively.

With respect to ROH length range, K14 exhibited a significantly higher number of ROH than either Q8 or K6 (Fig. 2C). Furthermore, the ROHs > 4 Mb in length were notably longer, on average, in K14 than K6 and Q8 (Fig. S1). Conversely, K6 displayed the highest proportion of short ROH (0-0.5 Mb) and the lowest proportion of long ROH (> 0.5 Mb) (Fig. 2D). The cumulative number of ROH on chromosomes 1–5 accounted for more than 60% of the total number of ROH, with K14 exhibiting the highest number of ROH fragments on chromosomes 1–9, 12, 13, 17, 18, 21, 23, 26, and 28, followed by Q8 and K6 (Fig. S2). Notably, chromosome 14 in K14 displayed the longest ROH with an average length of 1.49 Mb, followed by chromosome 7 in Q8 with an average length of 1.47 Mb (Fig. S3).

### Inbreeding coefficients estimation based on ROH

**Fig. 3:**
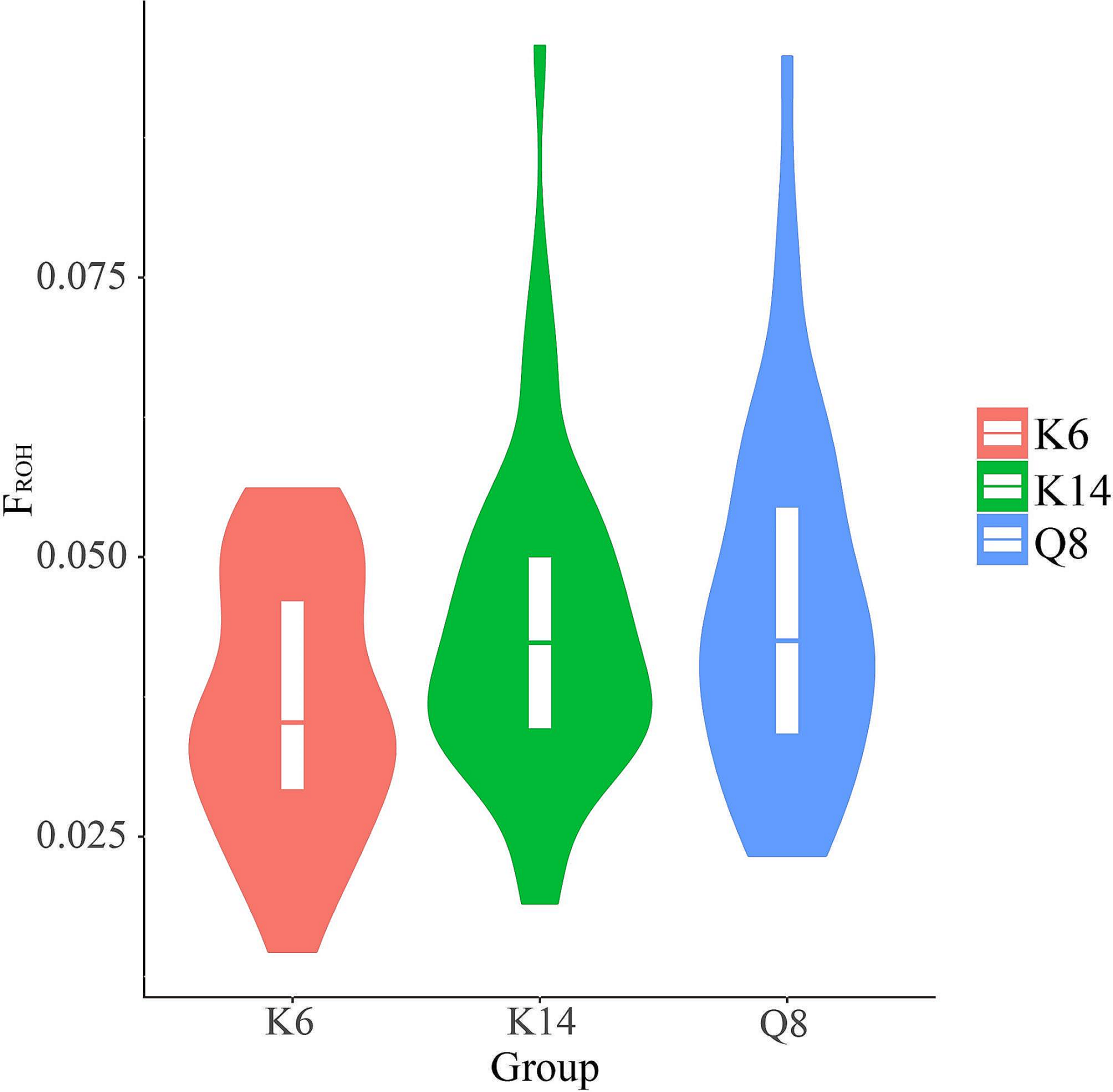
Distribution of inbreeding coefficients based on ROH for different Qingyuan partridge chicken populations

Inbreeding coefficients of the three Qingyuan partridge chickens were estimated using the ROH (*F*_*ROH*_) (Table 4). An average F_ROH_ value of 0.037 was calculated for K6, which was lower than those of K14 (0.043) and Q8 (0.045). Furthermore, *F*_*ROH*_ values for K6 showed the narrowest range of distribution (Fig. 3). Among all individuals, the highest *F*_*ROH*_ value was observed in K14 (0.096), followed by Q8 (0.095). The K6 population had the 19th highest individual *F*_*ROH*_ value, which was 0.056. Analysis of *F*_*ROH*_ statistics on various chromosomes revealed that chromosome 30 of K14 exhibited the highest average *F*_*ROH*_ value (0.624), followed by chromosome 38 of Q8 (0.502). Three additional chromosomes displayed average *F*_*ROH*_ values exceeding 0.3, specifically chromosome 30 of Q8 (0.490), chromosome 26 of K6 (0.302), and chromosome 25 of K6 (0.301). A comparison among the three populations studied revealed that, relative to Q8 and K14, population K6 exhibited lower average *F*_*ROH*_ values on 24 chromosomes (Fig. S4).

**Table 4:** Descriptive statistics for inbreeding coefficient (F) in each population

group	*F*_***ROH***_ (Mean ± SE)						*r*(*F*_***ROH***_, *F*)
	total	0-0.5	0.5-1	1–2	2–4	> 4	
K6	0.037 ± 0.011^a^	0.008 ± 0.002	0.009 ± 0.004	0.007 ± 0.003	0.007 ± 0.005^a^	0.006 ± 0.006^a^	0.49
K14	0.043 ± 0.014^b^	0.007 ± 0.002	0.009 ± 0.003	0.009 ± 0.004	0.008 ± 0.005^ab^	0.011 ± 0.011^b^	0.94
Q8	0.045 ± 0.015^b^	0.007 ± 0.002	0.010 ± 0.003	0.008 ± 0.004	0.010 ± 0.007^b^	0.010 ± 0.008^b^	0.70

*F*_ROH_, ROH-based inbreeding coefficient; r(*F*_*ROH*_,*F*), Correlation of ROH-based inbreeding coefficient with inbreeding coefficient F. Different lowercase letters indicate significant differences (*P* < 0.05) and no letter indicates non-significant differences (*P* > 0.05)

### Gene functional annotation

**Fig. 4:**
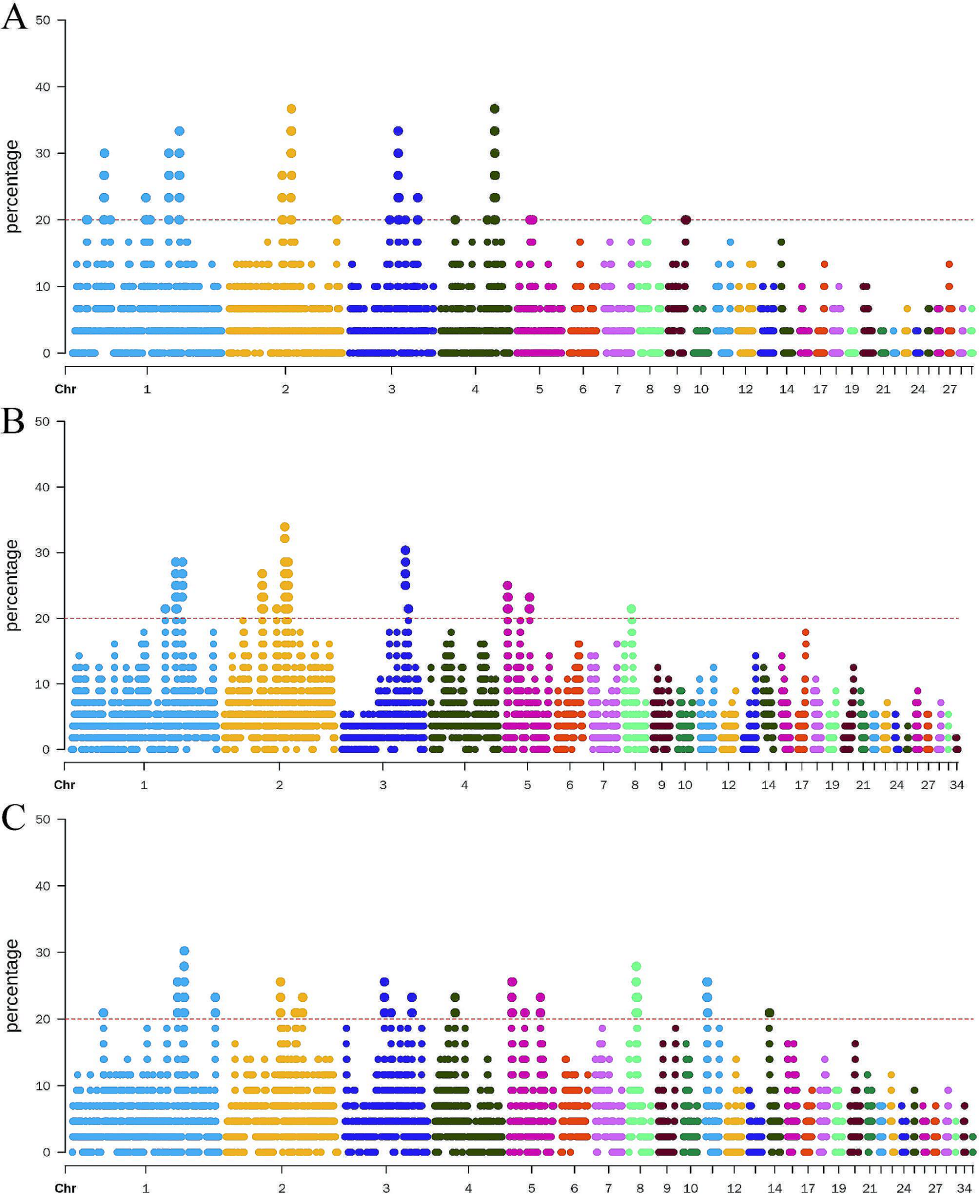
Manhattan plot of ROH frequency in different groups of Qingyuan partridge chickens. (**A**) K6. (**B**) K14. (**C**) Q8

Analyses were conducted to identify the genomic regions that were most associated with ROH regions in the three chicken populations and to show the frequency of each SNP within the ROH relative to their respective SNP positions along the chromosome (Fig. 4). The results showed that 56 ROH islands across all three populations, with 25 islands detected in K6, 13 in K14, and 18 in Q8. The average length distribution of these ROH islands ranged from 531.87 ± 486.78 to 727.38 ± 524.34 kb, and the average number of continuous SNPs within these islands ranged from 154.22 ± 107.61 to 299.24 ± 284.38.

Utilizing the established criteria for ROH island screening, eight ROH islands were identified and subsequently analyzed. All eight ROH islands were found to overlap with 17 QTL regions in the chicken QTL database, encompassing various aspects, such as production, health, and exterior (Table S1). Within this set, three ROH islands were revealed as potentially associated with the breed-specific characteristics of Qingyuan partridge chickens, whereas the remaining five were linked to growth traits. The three ROH islands associated with breed-specific characteristics harbored 21 candidate genes, including *GLI3*, *ANO5*, *BLVRA*, *EFNB2*, *SLC5A12*, and *SVIP*, while the five ROH islands related to growth traits also contained 21 candidate genes, including *IRX1*, *IRX2*, *EGFR*, *TPK1*, *NOVA1*, *BDNF* and so on (Table S2).

### Signatures of selection in ROH islands

**Fig. 5:**
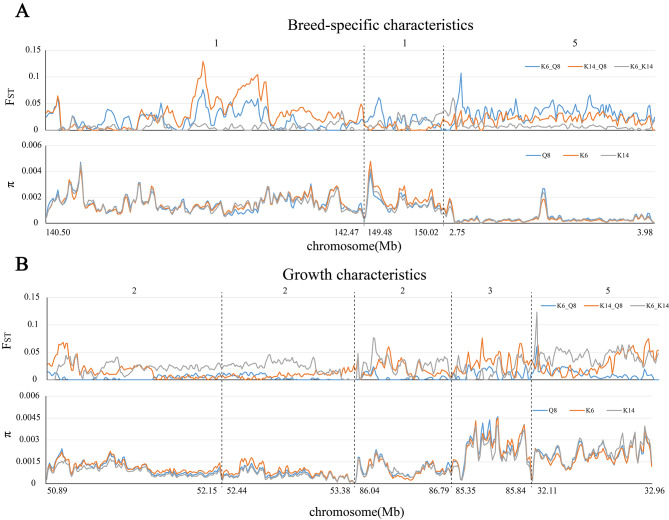
Selection signal analysis of ROH islands in different groups. (**A**) Breed-specific characteristics-related ROH islands; (**B**) Growth characteristic-related ROH islands

Subsequently, the genetic divergence of ROH islands between various populations was assessed using selection signals and nucleotide diversity analysis. In the case of ROH islands linked to breed-specific attributes, the K6 and K14 breeding populations exhibited selection signals in contrast to Q8, with K14 showing no divergence from K6 (Fig. 5A). Conversely, in the ROH islands associated with growth traits, the extensively bred K14 population showed selection signals when compared to the moderately bred K6 population and the preserved Q8 population (Fig. 5B).

## Discussion

The Qingyuan partridge chicken possesses superior germplasm characteristics, as noted in previous research [37]. The K-line selection population of Qingyuan partridge chickens is a breeding group primarily chosen for growth traits, with light variations in appearance emerging between the recently selected generations and the preserved population. Nonetheless, this selection procedures typically relies on phenotypic traits. Consequently, ongoing monitoring of populations within the same breed at various selection stages aids in enhancing genetic advancements and preserving germplasm resources. Whole-genome resequencing data have been established as a dependable method for identifying selection sweeps, yet recent research has predominantly concentrated on populations exhibiting substantial phenotypic disparities. This study endeavors to utilize ROH for genetic assessment and the identification of candidate genes associated with breed-specific traits and selected characteristics. Our investigation introduces a novel concept and methodology for the exploration of functional genes within distinct breeds.

The formation of ROH is primarily attributed to the inheritance of identical haplotypes from a shared ancestor, resulting in the preservation of pure genetic lines across generations [20]. The presence of a ROH island indicates potential genomic regions that experienced either natural or artificial selection [42]. The shortened length of the ROH island is likely due to a limited heterozygous gap in the ROH sequence [33]. This phenomenon has led to the identification of various genes linked to chicken growth and breed-specific traits. Analysis of the breed-specific characteristic region has revealed an overlap with selected regions in multiple chicken breeds [43–45], implying that genes within these regions may play a role in the development and manifestation of unique breed-specific traits across different chicken populations, mainly related to emergence and reproduction, such as the *FAM155A*, *ARGLU1*, and *LOC107050476* genes located on chromosome 1, and the *ANO5*, *BBOX1*, and *CCDC34* genes located on chromosome 5. Specifically, the *FAM155A* gene is associated with reproductive performance and immune response in sheep [46], and with daily weight gain in pigs [47]. The *ARGLU1* gene is important for stress hormone signaling and embryonic development, while the *LOC107050476* gene was found to regulate early embryonic development in blue-breasted quails. Further, the *ANO5* gene affects conversion and stress response in chickens. The *BBOX1* gene, which is highly expressed in commercial broilers, is mainly involved in regulating feed efficiency [48], and the *CCDC34* gene plays an important role in chickens adaptation [45]. In regions of ROH island associated with growth traits, a majority of genes were found to be linked to growth traits. Through a comparison of these genes with identified QTL, genes such as *NOVA1*, implicated in chicken abdominal fat formation [49], and a gene related to aromatic substances crucial in thiamine metabolism were discovered [50]. Additionally, genes associated with other traits were identified in this region, including *GLI3* for limb development [51], *BLVRA* for eggshell color [52], and *IRX1* and *IRX2* for chicken development [53, 54]. Moreover, the nucleotide diversity and *F*_*ST*_ values observed in the eight ROH island regions provide additional evidence that these regions have been influenced by selective pressures. These findings serve to reinforce the utility of ROH analysis in predicting regions under selection and monitoring the selection process in agricultural animal populations.

The assessment of genetic diversity is crucial for understanding the selection and conservation efforts within a breed, and elucidating the genetic diversity of a breed can aid in the preservation and utilization of its genetic resources. Notably, the genetic compositions of the three populations examined in this study exhibited significant differences, indicating that the Qingyuan partridge chicken population has significantly differentiated during the breeding or preservation processes. Specifically, when comparing the K14 and K6 populations, discernible differences were observed between male and female individuals within the latter group, suggesting an increase in population homogeneity during the selection process. Furthermore, the heightened homogeneity of the preserved population Q8 implies potential inbreeding or selective during the preservation process, ultimately leading to a decrease in genetic diversity within the preserved population. While inbreeding is a crucial component of breeding practices, it is essential to judiciously apply this process to the conservation and selection of local breeds. The findings of this study indicate that the trend of the inbreeding coefficient *F* in this study differed from that of the ROH-based inbreeding coefficient, with the latter demonstrating closer alignment with the selection history of Qingyuan partridge chickens. This consistency with prior research confirming the greater accuracy of ROH-based inbreeding coefficients suggests their superiority in evaluating the inbreeding status of a population [22, 23].

The characteristics of ROH, including number, length, frequency, distribution, and inbreeding coefficient, offer valuable insights for investigating demographic patterns of livestock species [35]. The genomic ROH of two breeding groups and one preserved population of Qingyuan partridge chickens were compared. The results revealed that the preserved population Q8 and the breeding population K14 exhibited similar levels of ROH, with the inbreeding coefficient suggesting that the inbreeding status Q8 was not significantly different from that of K14. Specifically, the length and frequency of long-segment ROH in Q8 were only slightly higher than those in K14, indicating a potentially higher inbreeding frequency in the preserved population in the recent generation, which was also confirmed by the relative aggregation of the Q8 population in PCA. The numbers of long-segment ROH above 4 Mb were less in the Qingyuan partridge chickens than those in commercial chickens [27] and Italian native chickens [55], suggesting less recent inbreeding in the Qingyuan partridge chickens. Moreover, the decreased inbreeding coefficient in Qingyuan partridge chickens serves as additional support for a reduced level of selection. The number of ROH islands in generations K6-K14 of the breeding population declined, while their length increased. This suggests that the longer ROH islands observed in K14 may be attributed to artificial selection. Similarly, the noticeable decrease in ROH islands in the Q8 group, which experienced weaker artificial selection, reinforces this finding.

## Conclusion

This study conducted genomic evaluation and ROH analyses for the first time on a multi-generation artificially selected chicken population and a preserved population. The findings suggest a low level of genomic linkage and degree of inbreeding within both bred and preserved populations, indicating abundant genetic diversity and adequate genetic potential of the Qingyuan partridge chicken. Throughout the long-term selection process, genes associated with growth traits and breed-specific characteristics of the Qingyuan partridge chicken have gradually accumulated within several ROH islands. The results of this study not only contribute to the evaluation of the inbreeding coefficient and breeding preservation status of Qingyuan partridge chickens, but also provide new insights for performing genetic assessment and identifying the genes of breed-specific or selected characteristics.

## Materials and methods

### Ethics statement

All experimental procedures used in this study were approved by the Laboratory Animal Welfare and Animal Experimental Ethical Inspection Board of Foshan University (No. 19,112,201).

### Chickens and sample collection

All three different populations of Qingyuan partridge chickens used in this study were fed according to the feeding and management standard of the National Breeding and Conservation Farm for Qingyuan partridge chickens. Therefore, both the preserved and breeding chickens were raised with the same dietary program and management procedures. In brief, chicks were kept on flat net during the brooding period and transferred to cage rearing with limited feeding procedure from the age of 42 days. The preservation population was kept from the original core flock by adopting the method of equal and random reservation of family lines, while the breeding population used in this study (K line) was directionally selected to improve growth performance from the original core flock of the breed by combining family selection and individual selection. The main selection traits included growth rate, premature sexual maturity, early plumage maturity, body shape, fertilization rate and population uniformity.

In this study, a total of 134 blood samples, representing the preserved population (Q8, ♂: 29 and ♀: 15), the sixth generation of breeding population (K6, ♂: 12 and ♀: 19) and the fourteenth generation of breeding population (K14, ♂: 29 and ♀: 30), were randomly collected from the National Breeding and Conservation Farm for Qingyuan partridge chickens. Whole blood samples were collected from the sub-wing vein and stored in EDTA anticoagulant tubes at − 80℃.

### DNA extraction, resequencing and SNP genotyping

Genomic DNA was extracted from blood samples using the Ezup Column Blood Genomic DNA Extraction Kit (Vazyme Biotech Co., Ltd, China) according to the kit instructions. The extracted DNA was analyzed for integrity using agarose gel electrophoresis, and DNA concentration and purity were determined using a NanoDrop. All qualified samples were immediately stored at − 20 °C and used for library construction (Paired-end, 2 × 150 bp). Whole-genome resequencing was performed using the BGI DNBSEQ-T7 platform at China National GeneBank (Shenzhen, China). The average sequencing depth for each sample was greater than 15×.

Raw reads containing adaptor sequences, low-quality bases > 10%, and N content > 10% were filtered using fastp (v0.20.0) [56]. The clean data were mapped to the most recent chicken reference genome (GRCg7b, GCF_016699485.2) using the Burrows-Wheeler Aligner (BWA v0.7.17) software [57] with default parameters. Single nucleotide polymorphisms (SNP) calling was performed by using the default parameters of the HaplotypeCaller module in GATK v3.5 software [58], and SNP filtration was performed using the VariantFiltration module in GATK, with the following values for filtration parameters: QD < 5.0, MQ < 40.0, FS > 60, MQRankSum < -12.5, ReadPosRankSum < -8.0, and 3 or more SNPs within 10 bp. Only autosomes were used for subsequent analysis to avoid the influence of sex chromosomes. PLINK v1.9 [59] software was used for further quality control of the SNP data, with parameters set as follows: (1) individuals with a call rate < 0.99 were removed (--mind 0.01); (2) SNP loci with SNP call rates less than 99% were discarded (--geno 0.01); (3) SNPs with minimum allele frequency (MAF) less than 0.05 were removed (--maf 0.05); (4) Hardy-Weinberg equilibrium *P* < 1 × 10 − 5 (--hwe 0.00001). The genomic coordinates of all obtained SNPs were based on GRCg7b.

### Population structure and genetic diversity analyses

After quality control, high-quality SNPs were subjected to the phylogenetic and population structure analyses. The identical-by-state matrix were computed using PLINK software with the parameter “--distance-matrix”, and then were applied to construct a neighbor-joining (NJ) tree via MEGA 11 [60]. The NJ tree was visualized and optimized using FigTree v14.4 (http://tree.bio.ed.ac.uk/software/figtree/). PCA was performed using PLINK with the parameters “--pca”, and the first 2 components were visualized using the ggplot2 package in R [61]. Several parameters were used to assess the genetic diversity of the three populations. The observed heterozygosity (Ho) and the expected heterozygosity (He) were calculated using the command “PLINK --hardy”. The linkage disequilibrium coefficient r^2^ and the decay distance were calculated using PopLDdecay software [62], and the results were plotted as linkage disequilibrium decay (LD decay) maps using the plot module built into the PopLDdecay software. The inbreeding coefficient (*F*) was calculated using PLINK with the parameter “--het”. The linked loci in the genomic data were removed using PLINK software with the parameter “--indep-pairwise 50 5 0.1”. The effective population size was estimated using SNeP v1.1 software [63] based on filtered data.

### ROH detection and inbreeding coefficient calculation

Linkage disequilibrium-filtered loci were used for ROH analysis to reduce the probability of detecting non-autologous ROH due to trait selection and to improve detection accuracy. The sliding window method was used to detect the ROH using the PLINK software. The detailed parameters are as follows: (1) The sliding windows of size was 100 SNPs; (2) the ROH fragments could allow up to two SNPs deletion and one heterozygous; (3) the minimum number of SNPs for a ROH was 50; (4) the minimum length of a ROH was 300 kb; (5) the minimum SNP density of a ROH was 1 SNP/25 kb; (6) the maximum interval between continuous homozygous SNPs was 100 kb, and (7) the threshold value for the sliding window was 0.05. The ROH results were then classified, counted, and the *F*_*ROH*_ value was calculated using the detectRUN package in R [64].

### Detection of the dissimilarities in ROH island

To identify high-frequency ROH in the genome, the SNP frequency in the ROHs was calculated by counting the number of times each SNP occurred in the ROH across individuals. In this study, a threshold of 20% was used to identify high-frequency regions of the ROH. Neighboring SNPs above the threshold were combined into a genomic region called the ROH island for further analysis. The high-frequency ROH regions in the three populations were screened according to the following two criteria to identify the characteristic genes of Qingyuan partridge chicken during breeding and preservation: (1) the regions in K14 with a greater length than in K6; (2) the area in K14 shared by Q8. In contrast, the regions with increased length that differed between K14 and Q8 were considered as most likely related to growth traits.

### Analysis and functional annotation of selection signatures in ROH island

To validate the selection signal of the ROH island obtained from screening, paired Fst values and genotype frequencies were evaluated for the three populations using PLINK software to further compare the selection signatures present in the ROH islands obtained from the selection screen. The results were visualized using the R. Then the candidate genes and regions were annotated using the NCBI database and the chicken QTLdb [65]. A comprehensive literature review was conducted to determine the biological function of each annotated gene.

### Statistical analyses

All data are shown as mean ± standard error (SE). Significance tests for differences between groups were performed by one-way analysis of variance (ANOVA) or T test for phenotypic data using SPSS 22.0 software. All values with *P* < 0.05 indicated a significant difference, and *P* < 0.01 indicated a highly significant difference.

### Electronic supplementary material

Below is the link to the electronic supplementary material.

Additional file 1: Fig. S1. Mean length of ROH in different length categories

Additional file 2: Fig. S2. The number of ROH per chromosome in different Qingyuan partridge chicken populations

Additional file 3: Fig. S3. The mean length of ROH per chromosome in different Qingyuan partridge chicken populations

Additional file 4: Fig. S4. Distribution of inbreeding coefficients based on ROH for each chromosome

Additional file 5: Table S1. Basic information about ROH Island for three groups

Additional file 6: Table S2. Genomic regions associated with breed characteristics and growth traits identified on ROH Island

## Data Availability

The datasets generated in this study have been deposited into the China National GeneBank DataBase (CNGBdb) repository under the accession number CNP0004838 (https://db.cngb.org/search/project/CNP0004838/).
