# Supplementary figures and images for: Genetic assessment and candidate genes identification for breed-specific characteristics of Qingyuan partridge chicken based on runs of homozygosity
Source: BMC Genomics. 2024 Jun 10;25:577. doi: 10.1186/s12864-024-10492-y (PMC11163754; doi:10.1186/s12864-024-10492-y)

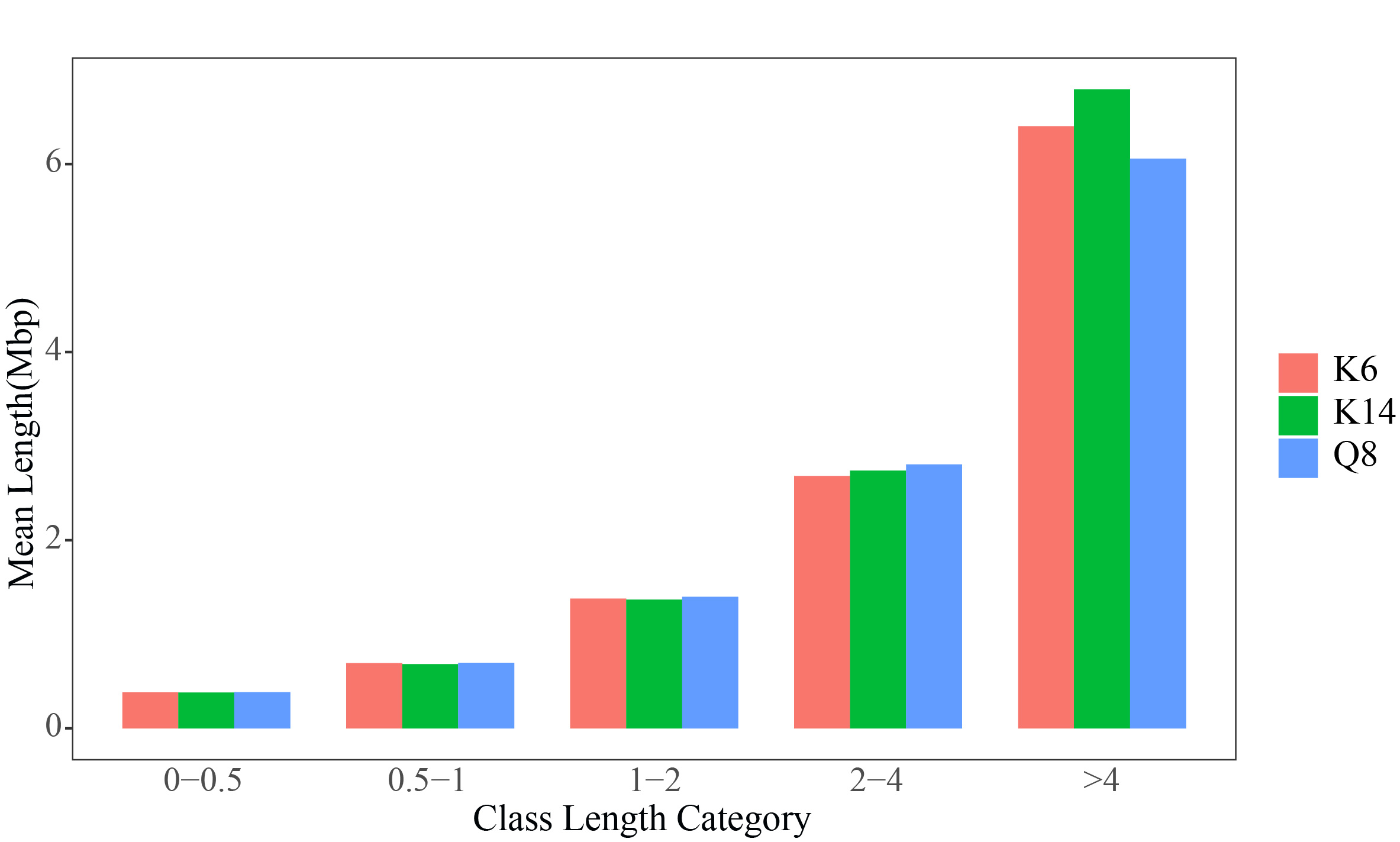

Supplement: Supplementary file 1 — Additional file 1: Fig. S1. Mean length of ROH in different length categories [file 12864_2024_10492_MOESM1_ESM.jpg]

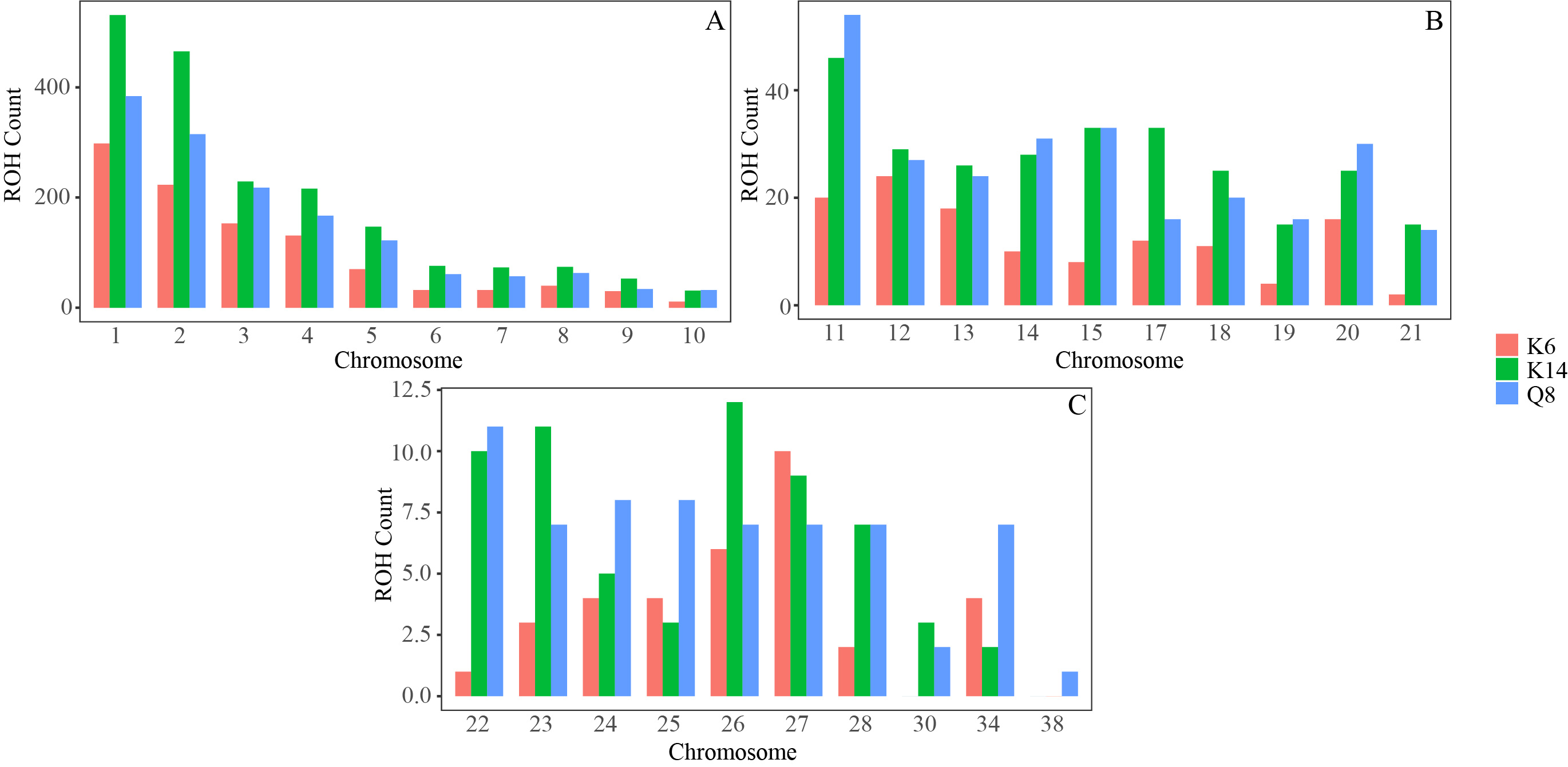

Supplement: Supplementary file 2 — Additional file 2: Fig. S2. The number of ROH per chromosome in different Qingyuan partridge chicken populations [file 12864_2024_10492_MOESM2_ESM.jpg]

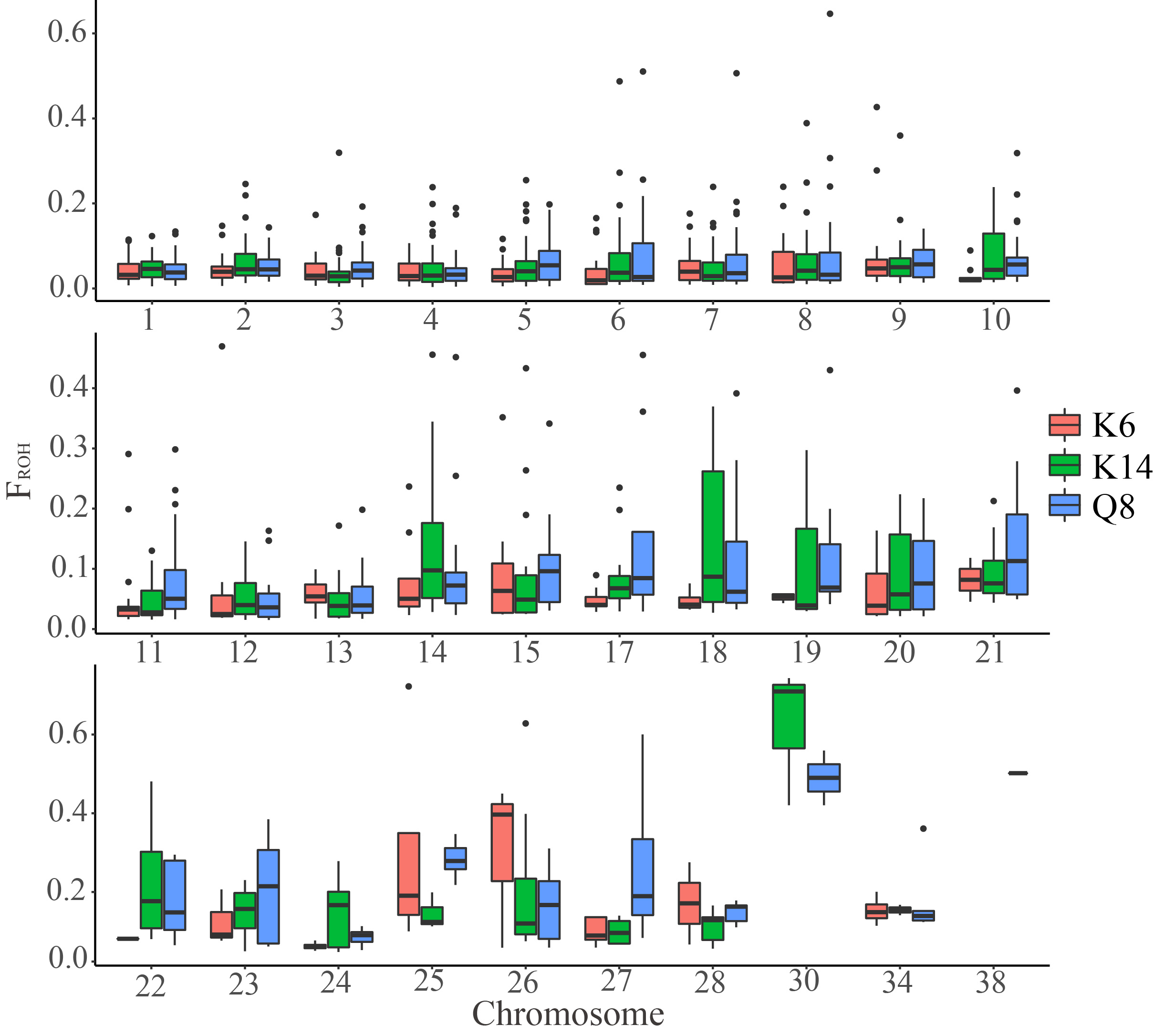

Supplement: Supplementary file 3 — Additional file 3: Fig. S3. The mean length of ROH per chromosome in different Qingyuan partridge chicken populations [file 12864_2024_10492_MOESM3_ESM.jpg]

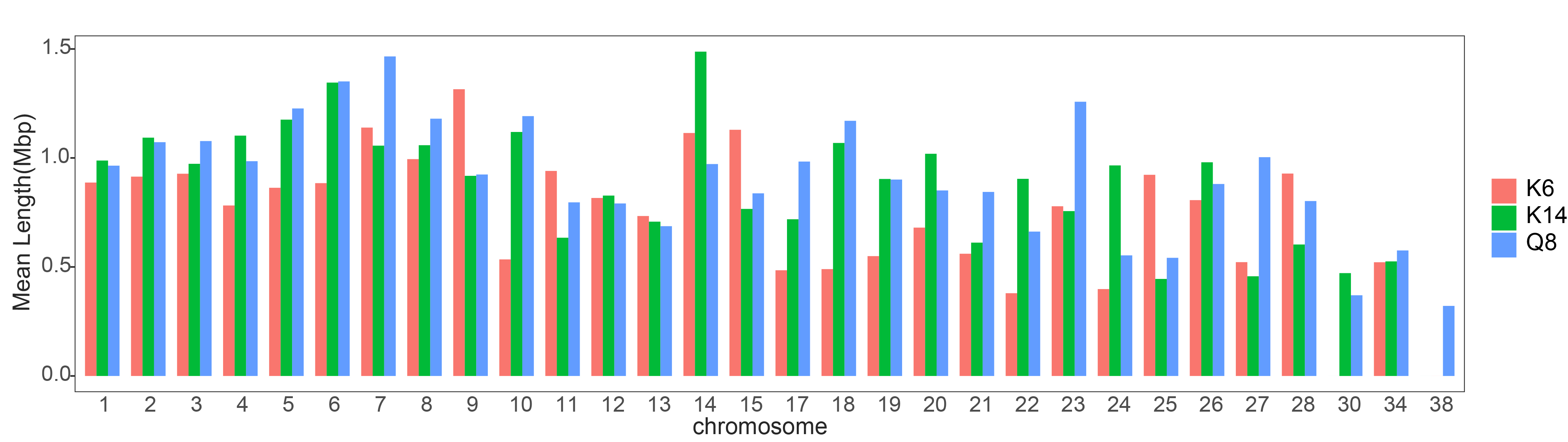

Supplement: Supplementary file 4 — Additional file 4: Fig. S4. Distribution of inbreeding coefficients based on ROH for each chromosome [file 12864_2024_10492_MOESM4_ESM.jpg]
